# Resilience and Risk Factors Predict Family Stress Among Married Palestinians in Israel During the COVID-19 Pandemic

**DOI:** 10.3389/fpsyg.2022.913330

**Published:** 2022-06-24

**Authors:** Niveen M. Hassan-Abbas

**Affiliations:** Department of Special Education and Psychology, Department of Behavioral Sciences, Zefat Academic College, Safed, Israel

**Keywords:** Palestinian families in Israel, COVID-19 pandemic, family resilience, individual resilience, risk factors, family stress

## Abstract

The present study examined effects of sociodemographic, risk, and resilience factors on marital, parental, and financial stress early in the COVID-19 pandemic. A cross-sectional online survey was conducted among 480 married Palestinians living in Israel, using self-report questionnaires. Descriptive statistics and bivariate correlations were computed. Then, hierarchical multiple regression analyses were conducted to predict each of the three stress measures. Finally, dominance analyses were conducted to compare the contributions of sociodemographic, risk, and resilience factors. The results showed considerable differences between predictors of marital and parental stress and predictors of financial stress. For marital and parental stress, resilience factors mainly family resilience were the strongest predictors, followed by risk factors and then sociodemographic characteristics. For financial stress, risk factors (mainly financial damage and prior stressful life events) were the strongest predictors, followed by sociodemographic characteristics and resilience factors (mainly individual resilience). These results suggest that family and individual resilience reduce marital and parental stress under difficult conditions, including the COVID-19 pandemic. Though resilience has been found to ameliorate financial stress to some extent, it appears that other measures are required to manage stress related to financial difficulties.

## Introduction

Like other acute and long-term crises, the COVID-19 pandemic has detrimentally affected well-being and functioning among both individuals and families (Arnoso Martínez and Eiroá-Orosa, 2010; Hoffman and Kruczek, 2011; Chang et al., 2015; Pietromonaco and Overall, 2021). Research has shown that pandemic-related stressors predicted a decline in mental health (Barzilay et al., 2020; Forte et al., 2020) and increases in relationship, family, and parental stress (Goldberg et al., 2021; Spinelli et al., 2021). These outcomes, in turn, have led to a range of negative implications for parents and children (Brown et al., 2020; Spinelli et al., 2021).

To assess the specific ways in which the COVID-19 pandemic might have impacted couple and family relationships, recent work (Chen and Bonanno, 2020; Prime et al., 2020; Pietromonaco and Overall, 2021) has drawn on earlier studies in the field. Many of these studies emphasize risk factors, such as the severity of exposure to trauma, and social or economic burden (Khamis, 1998; Eshel et al., 2019; James-Hawkins et al., 2019; Chen and Bonanno, 2020; Prime et al., 2020).

Other studies, however, discuss individual (Chen and Bonanno, 2020) and familial (Prime et al., 2020) resilience factors that operate as buffers against such risks. Research indeed shows that the vast majority of individuals and families demonstrate resilience following traumatic events like disease outbreaks (Chen and Bonanno, 2020). A broad systematic review conducted by Sousa et al. (2013) indicated that despite considerable adversity associated with political violence, individuals and communities showed considerable resilience. Indeed, it is known that family resources and protective factors help to determine how well parents adapt to health crises and moderate negative psychological outcomes, which in turn impact parental, family, and child well-being (Prime et al., 2020; Goldberg et al., 2021). Exploring these factors can help us understand family responses to crisis and subsequently guide support and intervention programs.

The salience of family and individual resilience for reducing stress has been broadly studied in the context of various types of stressful conditions, such as physical illness of a partner (Daniel et al., 2009; Li et al., 2018), significant financial difficulties (Amato and Rogers, 1997), and significant life events (Cohan, 2010), all of which constitute risk factors for marital dissatisfaction and divorce. Some research focuses specifically on the conditions that simultaneously create stress throughout communities, like disease outbreaks, natural disasters, and terrorist attacks. In such cases, the interplay between risk and resilience can differ from cases of individual- or family specific stressors, as broader circles are affected and external social support is often disrupted or overloaded (Cohan, 2010; Prime et al., 2020).

Going beyond the individual, family, and community stressors addressed in the existing literature, the COVID-19 pandemic created unprecedented conditions, justifying an in-depth examination of risk and resilience in coping with stressful events. COVID-19 concurrently brought on a range of challenges and difficulties related to job loss, financial changes, lack of childcare, and confinement or isolation due to social distancing, alongside significant concerns related to illness and health. For couples and families, these types of stressors often influence familial structure, routines, communication patterns, and beliefs (Prime et al., 2020). They can also affect the quality and stability of couple relationships and increase risk for adverse relationship outcomes (Goldberg et al., 2021; Pietromonaco and Overall, 2021).

Psychological resilience is defined as “the process of, capacity for, or outcome of successful adaptation despite challenging or threatening circumstances” (Masten et al., 1990, p. 426). At the individual level, resilience moderates stress-and-coping processes to reduce negative and enhance positive outcomes (Rutter, 1987, 1999; Carver, 1998). According to Pooley and Cohen (2010), resilience involves utilization of internal and external resources to enable resourcefulness, hope, and healing. Through this adaptation process, humans are able, under adverse circumstances, to meet short- and long-term goals in the face of various challenges (Lutha and Cicchetti, 2000; Anderson et al., 2011; Southwick et al., 2014). Resilience combines protective factors that modify, ameliorate, or alter responses to environmental stressors that predispose people to maladaptive outcomes (Bonanno et al., 2015). Studies have revealed several such protective resources that build resilience, among them self-esteem, optimism, sense of hope, coping ability, attitudes, and personality (Suedfeld, 2015; Galatzer-Levy et al., 2018; Eshel et al., 2019). The role of individual resilience has been investigated broadly in the context of political violence, trauma, natural disasters, and epidemics (Khamis, 1998; Chang and Sivam, 2004; Arnoso Martínez and Eiroá-Orosa, 2010; Sousa et al., 2013).

Alongside individual resilience, research has demonstrated the beneficial impact of significant relationships (Walsh, 1996). Going beyond the influence of individual family members or caregivers on individual resilience, a systemic perspective arose, focusing on risk and resilience in the family as a functional unit (Walsh, 1996, 2016). Systems theory presupposes that crises and persistent life challenges impact the whole family. Family resilience, then, facilitates positive adaptation among individual family members and strengthens them as a unit (Walsh, 2016). It is believed to incorporate solidarity, or the perception of within-family connectedness and unity, belief systems related to how the family defines and deals with adversity, regulation of negative emotions, fostering of positive outlooks, and spirituality (Chang et al., 2015; Walsh, 2015; Dalton et al., 2020). Thus, despite the friction and even disintegration associated with crisis or trauma, some families remain able to function.

Several studies have addressed family resilience in the context of epidemics and community health crises. Chang and Sivam (2004) attributed family differences in dealing with the severe acute respiratory syndrome (SARS) pandemic to family support. Another SARS study showed that in family dyads comprising a hospital nurse and a family member, the nurse’s subjective well-being was associated with her own perceived family resilience and with that of her partner (Neo et al., 2016). Chang et al. (2015) also investigated the role of family resilience among nurses dealing with external stressors while working with infected patients during a major epidemic in Singapore. They found that family resilience, like individual resilience, predicted subjective well-being.

### Risk and Resilience During COVID-19

Recent research has addressed both risk and resilience factors in the context of the COVID-19 pandemic, under the assumption that they interact in determining individual and family functioning (Barzilay et al., 2020; Kimhi et al., 2020b,c; Prime et al., 2020; Shanahan et al., 2020). Likewise, other studies have examined whether and how sociodemographic characteristics predict differences in coping during COVID-19 (Kimhi et al., 2020b,c; Goldberg et al., 2021; Spinelli et al., 2021).

Kimhi et al. (2020b) showed that older age and lower income prior to the pandemic were associated with a greater sense of danger and that women reported a greater sense of danger and distress than did men (Kimhi et al., 2020b). Education level had a strong, inverse correlation with anxiety symptoms (Kimhi et al., 2020c) and marital and parenting stress (Goldberg et al., 2021; Spinelli et al., 2021). In contrast, an Indian study found no significant associations between age, gender, education, and income on quality of life during the novel coronavirus crisis (Kharshiing et al., 2021).

Beyond the risks associated with sociodemographic variables, other risk factors found in the context of COVID-19 include worries related to virus exposure (Barzilay et al., 2020) and pandemic-related stressors including financial disruption, job loss, and suspension of educational programs (Shanahan et al., 2020). Perceived susceptibility, perceived severity, and anxiety regarding the pandemic were also related to lower quality of life (Kharshiing et al., 2021). These findings are in accordance with other work showing that financial burden following a pandemic significantly predicts generalized anxiety and depression (Barzilay et al., 2020) as well as sense of danger and distress (Kimhi et al., 2020c). Stressful life events in the period before the pandemic were also associated with higher risk of emotional distress (Shanahan et al., 2020).

Additional work shows that these types of risks have been counteracted by various resilience factors during the pandemic (Barzilay et al., 2020; Kimhi et al., 2020b,c; Prime et al., 2020; Shanahan et al., 2020). Barzilay et al. (2020) showed that high levels of resilience were associated with lower levels of worry, generalized anxiety, and depression among healthcare providers and other participants. Similarly, Kimhi et al. (2020c) found that five variables, namely well-being, individual resilience, community resilience, financial difficulties, and gender, could predict both level of distress and sense of danger, two indicators of the negative impact of COVID-19. After well-being, individual resilience was the best predictor, suggesting that it could be a buffer in the face of COVID-19-related stress.

Relating more specifically to family resilience during COVID-19, Prime et al. (2020) drew on previous research to hypothesize that pandemic-related adversity would impact individuals and families differentially, in accordance with other related factors in their lives (Masten and Narayan, 2012; Doom and Cicchetti, 2020). Applying Walsh’s (2015) family resilience framework to the COVID-19 crisis, Prime and colleagues proposed that all families would mitigate, as best they could, to the amount and type of disruption they experienced. They concluded that high levels of family resilience would enable better coping and hardiness during this unprecedented time. In another study, Kharshiing et al. (2021) found that solidarity and identification with family members were positively associated with quality of life during the COVID-19 pandemic.

### The Current Study

The current study examined potential effects of sociodemographic characteristics, risk factors, and resilience factors on the experience of marital, parental, and financial stress during the COVID-19 pandemic. The model of conservation of resources (Hobfoll, 1989, 1998) posits that people strive to retain, protect, and build resources, and that they are threatened by the potential or actual loss of these resources. On the basis of this theory, the current study examined the assumption that the existence and maintenance of key personal, social, and material resources during and after the pandemic would be major predictors of lower family stress.

Studies that have investigated the interplay between these variables have found resilience factors to be the best predictors of outcomes in the context of various threats and adversities (Khamis, 1998; Sousa et al., 2013; Haque et al., 2015; Walsh, 2015; Eshel et al., 2019), including the COVID-19 pandemic (Kimhi et al., 2020b,c; Shanahan et al., 2020; Kharshiing et al., 2021). In an attempt to extend these findings beyond individual distress levels, the current study addressed family and relationship variables as well.

Specifically, the study examined two types of familial stress: marital and parental. These are two distinct constructs, measured by separate tools. Marital stress involves the quality of marital communication, spouse psychological and physiological well-being, and the time spouses spend together (Bodenmann, 2000; Bodenmann et al., 2007). Parental stress refers to the parent’s perception of mismatch between the resources available to fulfill parental roles and the demands of parenting (Abidin, 1992; in Spinelli et al., 2021).

Furthermore, the study specifically addressed the Palestinian population in Israel, which has been largely overlooked in research on crisis-management in general and in the context of the COVID-19 pandemic specifically. Culture and environment are key factors in determining how individuals and couples respond and adapt to stress (Huston, 2000). Palestinian society is generally considered collectivistic, a cultural pattern involving a primary sense of commitment to family and community. Collectivism is often reflected in a strong desire to fulfill the basic human needs and desires of family and community members, which yields a sense of personal satisfaction and self-actualization and a sense of living harmoniously with one’s collective (Haj-Yahia, 2019).

Families are indeed a significant source of support in Arab culture, and family members are often cited as partners in coping with daily problems and environmental stressors and as providers of mental health care (Khamis, 1998; Azaiza, 2013; Haque et al., 2015; Haj-Yahia, 2019). Similar patterns have been shown in traditionally collectivistic, family oriented Asian cultures, in which family support and solidarity are vital to the coping processes of individuals facing major life threats and adversity (e.g., China, India, Malaysia: Bentelspacher et al., 1994; Singapore: Chang and Sivam, 2004; Chang et al., 2015; Neo et al., 2016). Specifically, Palestinian society in Israel has undergone a continual process of modernization. At present, this cultural group tends to give precedence to the nuclear over the extended family (Azaiza, 2013), with the spouse (as opposed to extended family members) often serving as the individual’s main source of support (Azaiza, 2008), and increasingly greater reciprocity between wives and husbands in the marital relationship (Hassan-Abbas and Ronen, 2022). This suggests that resilience in the nuclear family is of particular importance, particularly when extended family support is limited by the circumstances, as in the COVID-19 pandemic. It is possible that the cultural emphasis on family would result in a more significant role for familial resilience, as compared to individual resilience, in predicting stress.

It is also noteworthy that at 21% of the total population, Palestinians in Israel constitute a minority group (Israel Central Bureau of Statistics, 2019)^1^. While the current study did not compare the Palestinian minority to the Jewish majority, potential effects of Israel’s sociopolitical circumstances should be considered. Minority ethnic groups in various countries have been found to experience greater difficulties than do majority groups, in various life areas (Greer and Chwalisz, 2007; Sewell, 2016; Kimhi et al., 2017; Braun-Lewensohn et al., 2021). Due to these continual difficulties, minorities are often found to have less resilience and coping resources (Greer and Chwalisz, 2007; Kimhi et al., 2017), making them more susceptible to stressors such as illness and disaster (Catz et al., 2002; Weems et al., 2010). Studies have shown that specifically during the COVID-19 pandemic, minorities including Arab groups reported elevated levels of stress, as compared to their respective majority groups (Garcia et al., 2021; Miconi et al., 2021).

Due to sociopolitical factors characterizing Israel, Palestinians living there are subject to various forms of discrimination and economic disadvantages (Keinan and Bar, 2007; Abu-Kaf, 2019) that are likely to affect stress and resilience levels. Indeed, researchers have found higher levels of psychological distress among Palestinians in Israel as compared to the Jewish majority (e.g., Baron-Epel et al., 2010; Abu-Kaf, 2019), as well as lower resilience factors including sense of coherence (Braun-Lewensohn and Sagy, 2011; Abu-Kaf et al., 2017) and individual, community, and national resilience (Marciano et al., 2020). Similar results were found in notably stressful political times (Gelkopf et al., 2008; Hobfoll et al., 2009; Kimhi et al., 2017) and, more recently, specifically during the COVID-19 pandemic (Kimhi et al., 2020a; Braun-Lewensohn et al., 2021). Still, it should be noted that some studies have shown a different trend, with Arab participants showing a higher sense of coherence (Marciano et al., 2020) and comparable individual resilience (Kimhi et al., 2020a) to Jewish participants.

### Research Questions and Hypotheses

The study examined the extent to which marital, parental, and financial stress were correlated with, and predicted by, sociodemographic characteristics (gender, age, religiosity, marriage duration, number of children, level of education, and average family income before the pandemic), risk factors (financial damage due to the pandemic, participant job loss, spouse job loss, exposure to COVID-19, and stressful life events in the 12 months prior to the pandemic); and individual and family resilience. For each of the stress variables, the study further aimed to determine which of the factors were stronger predictors.

The following hypotheses were examined: (1) sociodemographic characteristics would significantly predict all three types of stress; (2) risk factors would positively predict the three stress variables, beyond the effects of sociodemographic characteristics; (3) the two resilience factors (individual and family) would negatively predict the three stress types, beyond the effects of sociodemographic characteristics and risk factors; (4) resilience factors would better predict marital, parental, and financial stress, as compared with sociodemographic characteristics and risk factors.

Given the lack of previous studies comparing the contributions of individual and family resilience among Arab families dealing with crises in general and COVID-19 in particular, the study further examined whether family resilience or individual resilience would better predict stress.

## Materials and Methods

### Participants and Procedure

Data for this study were taken from a larger survey on family coping during the COVID-19 pandemic among Palestinian married women and men in Israel. A total of 502 heterosexual married adults from different regions in Israel filled out the questionnaire. Of these, 22 were omitted because they stopped in the middle (did not reach the final part of the questionnaire), leaving 480 participants. The sample size was determined *a priori* using G*Power (Faul et al., 2009), which determined that the minimum required sample size for multiple linear regression was 440 participants for a small effect size (0.025; α = 0.05) with 85% power.

Participants were asked through social media to participate in an online survey. To be included, participants had to be married and living with their spouse (divorced, separated, and widowed individuals were excluded), have at least one child, and have access to a smartphone, personal computer, or laptop and an Internet connection. The survey was distributed through a broad range of channels and platforms in an attempt to obtain participants with a broad sociodemographic range, with respect to age, gender, years of marriage, number of children, and socioeconomic status. Sociodemographic and risk factors of the final sample are presented in Table 1.

**TABLE 1:** Sample sociodemographic characteristics: descriptive statistics (*N* = 480).

Variable	Range and rating scale	Frequency	Percent	*M*	*SD*
Age	18–29	89	18.5	38.31	9.66
	30–39	190	39.6		
	40–49	143	29.8		
	50 +	58	12.1		
Gender	1. Male	141	29.4		
	2. Female	339	70.6		
Religion	1. Muslim	353	73.5		
	2. Christian	34	7.1		
	3. Druze	91	19		
Religiosity	1. Secular	43	9		
	2. Conservative	256	53.3		
	3. Religious	171	35.6		
	4. Very religious	6	1.3		
Marriage duration	1–54	—	—	14.73	9.48
Number of children	1–10	—	—	2.82	1.34
Education	1. Up to 12 school years	53	11		
	2. High school	93	19.4		
	3. Post-secondary education	42	8.8		
	4. Bachelor’s degree	163	34		
	5. Master’s degree	93	19.4		
	6. Ph.D.	14	2.9		
Participant employment	1. Unemployed	74	15.4		
	2. Employed	273	56.8		
	3. Self-employed	82	17.1		
	4. Student	19	4		
	5. Pensioner	3	0.6		
	6. Soldier	5	1		
	7. Other	24	5		
Spouse employment	1. Unemployed	60	12.5		
	2. Employed	246	51.2		
	3. Self-employed	123	25.6		
	4. Student	9	1.9		
	5. Pensioner	9	1.9		
	6. Soldier	10	2.1		
	7. Other	23	4.8		
Average family income before the pandemic	1. Much less than the average household income	75	15.6		
	2. Less than this amount	83	17.3		
	3. Equal to this amount	162	33.8		
	4. More than this amount	122	25.4		
	5. Much more than this amount	38	7.9		
Financial damage due to the pandemic	1. Not at all	122	25.4		
	2. A little	139	28.8		
	3. Somewhat	119	24.9		
	4. Quite a bit	63	13.2		
	5. A great deal	37	7.7		
Participant’s employment status during the pandemic	1. I continued to work as usual	120	25.2		
	2. I continued to work from home	102	21.4		
	3. I continued some of my work as usual and some from home	48	10.1		
	4. I was fired	36	7.5		
	5. I was sent on unpaid leave	82	17.2		
	6. Other	89	18.7		
Spouse’s employment status during the pandemic	1. S/he continued to work as usual	180	37.5		
	2. S/he continued to work	62	12.9		
	from home				
	3. S/he continued some of the work as usual and some from home	38	7.9		
	4. S/he was fired	40	8.3		
	5. S/he was sent on unpaid leave	95	19.8		
	6. Other	63	13.1		
Infection with the coronavirus	1. Me	4 yes, 476 no	8, 99.2		
	2. A family member	7 yes, 473 no	1.5, 98.5		
	3. A friend or acquaintance	59 yes, 421 no	12.3, 87.7		
	4. A town resident	369 yes, 111 no	76.9, 23.1		
Death by the coronavirus	1. A family member	5 yes, 457 no	1, 95.2		
	2. A friend or acquaintance	11 yes, 452 no	2.3, 94.2		
	3. A town resident	59 yes, 405 no	12.3, 84.4		
Quarantine due to the coronavirus	1. Me	65 yes, 415 no	13.5, 86.5		
	2. A family member	61 yes, 419 no	12.7, 81.3		
	3. A friend or acquaintance	136 yes, 344 no	28.3, 71.7		
	4. A town resident	351 yes, 129 no	73.1, 26.9		
Exposure to stressful life events 12 months prior to COVID-19	0. No exposure	108	22.5		
	1. Exposure to 1 domain	164	34.2		
	2. Exposure to 2 domains	86	17.9		
	3. Exposure to 3 domains	53	11.0		
	4. Exposure to 4 domains	29	6.0		
	5. Exposure to 5 domains	20	4.2		

The COVID-19 pandemic began to spread in Israel in January 2020. Between March 15 and May 7, 2020, the Israeli government implemented lockdown policies of varying degrees, initially requiring the entire population, with the exception of essential service providers, to leave their homes only for necessary provisions, and becoming less strict over time. Data for this study were collected just after the general lockdown was lifted (May 2020), at which point the public was gradually allowed to resume routine functioning. The participants were asked to complete self-report questionnaires that addressed their coping and stress since the outbreak of the coronavirus pandemic, including the first lockdown.

Data were collected following approval of the ethics committee of the academic institute where the study was conducted. Participants received a brief explanation regarding the study and were assured that anonymity and confidentiality would be maintained. All participants signed an informed consent form before completing the study questionnaire.

### Measures

As part of the larger study, participants completed a self-administered multi-component questionnaire addressing a range of family related issues. The items and scales used in the current study are described below.

### Family Resilience

An adapted, Arabic version of the Family Resilience Scale (Chang et al., 2015) was employed (see details on translation and adaptation below), including 18 of the original 21 items to assess how individual participants perceived their familial ability to cope with adversity. The scale comprises four subscales: (1) family solidarity (9 items); (2) meaning-making (4 items); (3) religiosity (2 items); and (4) emotional regulation (3 items). Family solidarity addresses within-family connectedness and unity as perceived by individual participants (e.g., “We are united”; “We do not give up for the sake of one another”; “We are practical in dealing with the situation”). Meaning-making taps into the family belief system, addressing how the family defines and deals with adversity (e.g., “We redefine the meaning of the situation”; “We make the situation as normal as possible”). Religiosity measures the family’s adherence to religious beliefs in dealing with stressful situations (e.g., “We pray to God”). Emotional regulation assesses regulatory efforts regarding emotional expressions within the family (e.g., “We control negative emotions”). Respondents were asked to indicate to what extent they agreed or disagreed with each statement, using a 5-point Likert scale ranging from 1 (strongly disagree) to 5 (strongly agree). A mean score was calculated for each sub-scale and for the total score. Higher scores indicate greater resilience in the family, as perceived by the individual. Cronbach’s alpha for the present study ranged from 0.73 to 0.90 across the four subscales. Cronbach’s alpha for the total score was 0.90.

The family resilience measure was translated into Arabic based on the English version. In the first phase, the questionnaire was translated into Arabic by the author. In the second stage, another professional in the field was asked to compare the Arabic version with the English version, to assess the questionnaire with respect to overall clarity and the clarity of specific words and instructions. In the third stage, the author prepared the final Arabic version of the questionnaire based on comments received from the judge. In addition, three items that are less relevant to Arab society were removed: “we pray to our ancestors for help,” “we believe that our ancestors will look after us,” and “we have a pillar in the family and we follow his leadership.”

### Individual Resilience

The 10-item Connor–Davidson Resilience Scale [CD-RISC 10; Campbell-Sills and Stein (2007)] was used to assess individual resilience. The measure was translated to Arabic using the same method described for the family resilience measure above. The scale comprises 10 items (e.g., “I am able to adapt when changes occur”; “I can deal with whatever comes”) extracted from the original 25-item CD-RISC (Connor and Davidson, 2003). Each item is rated on a 5-point Likert scale from 0 (not true at all) to 4 (true nearly all the time). A mean score ranging from 0 to 4 was calculated for all responses, with a higher mean reflecting greater resilience. In the current study, Cronbach’s alpha was 0.90.

### Risk Factors

Previous research has revealed several risk factors in the context of COVID-19, which are commonly reported in countries across the world (Barzilay et al., 2020; Shanahan et al., 2020) including Israel (Kimhi et al., 2020b,c). In the current study, the author chose to examine four risk categories that were addressed in the clear majority of these studies, in addition to one risk factor related to pre-pandemic social stressors (e.g., stressful life events). The risk factors examined were as follows:

(1) Financial damage due to the pandemic, measured by one question: Did your family’s financial situation worsen during the COVID-19 pandemic? Responses ranged from 1 (not at all) to 5 (a great deal).
(2) Negative effects on participant employment or working conditions, measured by one question: How would you describe your employment status during the COVID-19 pandemic and the lockdown? One of six responses was possible: 1. I continued to work as usual; 2. I continued to work from home; 3. I continued some of my work as usual and some from home; 4. I was fired; 5. I was sent on unpaid leave; 6. Other. All participants who marked “other” were not employed before the pandemic (unemployed, student, pensioner, soldier). Responses of 1, 2, 3, and 6 were coded as 0 (employment status unchanged); responses 4 and 5 were coded as 1 (job loss).
(3) Negative effects on spouse’s employment or working conditions, measured by one question: How would you describe your spouse’s employment status during the COVID-19 pandemic and the lockdown? One of six responses was possible: 1. S/he continued to work as usual; 2. S/he continued to work from home; 3. S/he continued some work as usual and completed some from home; 4. S/he was fired; 5. S/he was sent on unpaid leave; 6. Other. All participants who marked “other” had spouses who were not employed before the pandemic (unemployed, student, pensioner, soldier). Responses 1, 2, 3, and 6 were coded as 0 (employment status unchanged); responses 4 and 5 were coded as 1 (job loss).
(4) Exposure to COVID-19, measured by yes/no questions in three categories: 1. COVID-19 infection of the participant, a family member, a friend or acquaintance, or a town resident; 2. COVID-19-related death of a family member, a friend or acquaintance, or a town resident; 3. COVID-19-related quarantine of the participant, a family member, a friend or acquaintance, or a town resident. A total exposure score ranging from 0 to 11 was calculated by summing the positive responses in all categories.
(5) Stressful life events 12 months prior to COVID-19 measured based on Sarason et al.’s (1978) life experiences survey. Five yes/no questions were asked regarding potentially stressful life events in five domains during the 12 months prior to the outbreak of the COVID-19 pandemic. The questions addressed the following domains: personal injury (e.g., severe illness, handicap, mental health difficulties, accident); injured relative (e.g., severe illness, handicap, mental health difficulties, accident); experiences of loss (e.g., death of a loved one or relative); professional life events (e.g., participant’s unemployment, spouse’s unemployment, financial difficulties); and, severe relationship stress (social tension with relatives, friends, neighbors, or colleagues at work). Positive responses were summed to reach a total score ranging from 0 to 5.

### Stress Related to Marriage, Parenting, and Finances

A questionnaire compiled by Pearlin and Schooler (1978); translated to Arabic by Abboud (2002) was used to examine three areas of stress: marital (9 items, e.g., “When you think of the pleasures and problems of your daily life with your spouse, how frustrated do you feel?”); parental (7 items, e.g., “When you think of your experiences as parent, how worried do you feel?”); and financial stress (8 items, e.g., “When you think of your financial situation, how insecure do you feel?”). The 24 items were rated on a Likert scale ranging from 1 (“not at all”) to 4 (“very much”). The questions were adapted to address the COVID-19 pandemic and lockdown period. A mean score was calculated for each area, with higher scores indicated greater reported stress. Cronbach’s alpha values were 0.90, 0.91, and 0.91 for marital, parental, and financial stress, respectively.

### Sociodemographic Variables

The following sociodemographic attributes were examined: gender, age, religion, religiosity, place of residence, marriage duration, number of children, level of education, employment, and average family income before the pandemic.

### Statistical Analysis

The following analyses were conducted using R 4.0.2 (The R Foundation, 2022).^2^ The percentage of missing data was 1.03%, completely at random. Missing values were handled using imputation, the process of replacing missing data with regression values. First, descriptive statistics (means and standard deviations) were calculated and bivariate correlations were computed for the resilience and stress measures (see Table 2). Second, bivariate correlations were computed for the sociodemographic characteristics, risk factors, and stress and resilience measures (see Table 3). Third, hierarchical multiple regression analyses were conducted to predict each of the three stress measures (see Table 4). These analyses examined the extent to which risk factors and resilience factors explained the variance in each of the three stress measures, beyond the variance explained by sociodemographic variables. Hence, each of the three stress measures was regressed on the predictor variables. The following three blocks of the predictors were entered into the regression and multiple regression formulas: 1. sociodemographic variables, 2. risk factors, and 3. resilience factors. Finally, dominance analyses were conducted to determine the resilience factor with the greatest contribution to stress levels and to compare the contributions of sociodemographic, risk, and resilience factors (see Table 5). Dominance analysis is used to compare the relative importance of predictors in multiple regression models (Budescu, 1993; Azen and Budescu, 2003) and has been widely applied in previous research (e.g., Tighe and Schatschneider, 2014; Jiang et al., 2016). To apply this method, the R^2^ values of each independent variable are calculated across every possible model. The mean value of R^2^ represents the importance of this variable in the linear model.

**TABLE 2:** Resilience factors and stress measures: correlations and descriptive statistics (*N* = 480).

Variables	Marital stress	Parental stress	Financial stress	Individual resilience	Family resilience
Marital stress^a^					
Parental stress^a^	0.69**				
Financial stress^a^	0.55**	0.53**			
Individual resilience^b^	−0.34**	−0.32**	−0.29**		
Family resilience^c^	−0.40**	−0.34**	−0.20**	0.33**	
M	1.75	1.67	1.93	2.82	4.2
SD	0.64	0.65	0.72	0.74	0.5

^a^Mean scores could range from 1 for low stress to 4 for high stress.^b^Mean score could range from 1 for low levels of individual resilience to 5 for high levels of individual resilience. ^c^Mean scores could range from 0 for low levels of family resilience to 4 for high levels of family resilience. **p < 0.01.

**TABLE 3:** Sociodemographic characteristics, risk factors, resilience factors, and stress measures: correlations (*N* = 477).

Variables	Age	Gender^a^	Religiosity^b^	Education^c^	Marriage duration^d^	Number of children^e^	Average family income before the pandemic^f^	Financial damage due to the pandemic^g^	Participant’s job status during the pandemic^h^	Spouse’s job status during the pandemic^i^	Exposure to COVID-19^j^	Prior stressful life events^k^
Marital stress	0.02	0.06	–0.05	–0.08	0.07	0.08	−0.14**	0.26**	0.07	0.10*	0.00	0.15**
Parental stress	–0.01	0.06	0.00	–0.08	0.03	0.13**	−0.12*	0.25**	0.05	0.14**	0.03	0.16**
Financial stress	–0.05	0.04	–0.02	−0.15**	0.01	0.05	−0.25**	0.52**	0.16**	0.17**	–0.01	0.23**
Individual resilience	0.16**	–0.07	–0.06	0.15**	0.07	0.03	0.16**	−0.16**	–0.08	−0.10*	0.02	−0.11*
Family resilience	–0.05	0.07	0.05	–0.09	–0.05	–0.06	–0.01	–0.07	0.08	–0.01	0.04	–0.08

*^a^Gender: 0 = male (n = 141), 1 = female (n = 336). ^b^Religiosity: scores could range from 1 for secular to 4 for very religious. ^c^Education: scores could range from 1 for low education to 5 for high education. ^d^Marriage duration range from 1 to 54. ^e^Number of children range from 1 to 10. ^f^Average family income before the pandemic: scores could range from 1 for very low income to 5 for very high income. ^g^Financial damage due to the pandemic: scores could range from 1 for no damage to 5 for great damage. ^h^Participant’s job status during the pandemic: 0 = job status unchanged (n = 359), 1 = job loss (n = 118). ^i^Spouse’s job status during the pandemic: 0 = job status unchanged (n = 342), 1 = spouse’s job loss (n = 135). ^j^Exposure to COVID-19: scores could range from 0 for no exposure (infection, death, and quarantine) to 11 for high exposure. ^k^Prior stressful life events: scores could range from 0 for no exposure to 5 for high exposure.*

A correlation of 0.5 or higher was considered strong, 0.1–0.3 was considered moderate, and smaller than 0.1 was considered weak. *p < 0.05, **p < 0.01.

**TABLE 4:** Summary of hierarchical multiple regression results for predicting marital stress, parental stress, and financial stress (*N* = 477).

Blocks of predictors	Variable	Marital stress		Parental stress		Financial stress	
		β	t	β	t	β	t
1^st^ block: Sociodemographic characteristics	Education^a^	0.028	–0.56	–0.006	–0.13	–0.007	–0.14
	Number of children^b^	0.077	1.70^†^	0.120	2.63**	0.046	1.04
	Average family income before the pandemic^c^	–0.167	–3.40**	–0.121	–2.46*	–0.266	–5.54***.
	**R^2^ (**Δ** R^2^)**	0.031 (0.031**)		0.030 (0.030**)		0.075 (0.075***)	
2^nd^ block: Risk factors	Financial damage due to the pandemic^d^	0.249	4.67***	0.200	3.73***	0.490	10.37***
	Participant’s job status during the pandemic^e^	–0.013	–0.27	–0.022	–0.46	0.001	0.02
	Spouse’s job status during the pandemic^f^	–0.008	0.16	0.053	1.08	–0.038	–0.87
	Prior stressful life events^g^	0.086	1.84^†^	0.113	2.40*	0.106	2.55*
	**R^2^ (**Δ** R^2^)**	0.092 (0.060***)		0.086 (0.056***)		0.286 (0.211***)	
3*^rd^* block: Resilience factors	Individual resilience^h^	–0.191	–4.40***	–0.200	–4.47***	–0.164	–3.98***
	Family resilience^i^	–0.324	–7.54***	–0.257	–5.80***	–0.115	–2.82*
	**R^2^ (**Δ** R^2^)**	0.269 (0.177***)		0.222 (0.136***)		0.337 (0.051***)	

^a^Education: scores could range from 1 for low education to 5 for high education. ^b^Number of children range from 1 to 10. ^c^Average family income before the pandemic: scores could range from 1 for very low income to 5 for very high income. ^d^Financial damage due to the pandemic: scores could range from 1 for no damage to 5 for great damage. ^e^Participant’s job status during the pandemic: 0 = job status unchanged (n = 359), 1 = job loss (n = 118). ^f^Spouse’s job status during the pandemic: 0 = job status unchanged (n = 342), 1 = spouse’s job loss (n = 135). ^g^Prior stressful life events: scores could range from 0 for no exposure to 5 for high exposure. ^h^Mean score could range from 1 for low levels of individual resilience to 5 for high levels of individual resilience. ^i^Mean scores could range from 0 for low levels of family resilience to 4 for high levels of family resilience. ΔR^2^ = R^2^ change. ^†^p < 0.10, *p < 0.05, **p < 0.01, ***p < 0.001.

**TABLE 5:** Summary of dominance analysis results: means of prediction variance for each dependent variable (*N* = 477*).*

	Marital stress	Parental stress	Financial stress
Family resilience^a^	12.5%	8.5%	2.3%
Individual resilience^b^	6.3%	6.3%	4.4%
Financial damage due to the pandemic^c^	3.8%	3.3%	20%
Prior stressful life events^d^	1%	1.3%	2.2%
Participant’s job status during the pandemic^e^	0.2%	0.1%	1%
Spouse’s job status during the pandemic^f^	0.4%	0.8%	1.1%
Average family income before the pandemic^g^	0.6%	0.4%	0.2%
Education^h^	0.2%	0.2%	0.6%
Number of children^i^	0.5%	1.2%	0.2%

*^a^Mean scores could range from 0 for low levels of family resilience to 4 for high levels of family resilience. ^b^Mean score could range from 1 for low levels of individual resilience to 5 for high levels of individual resilience. ^c^Financial damage due to the pandemic: scores could range from 1 for no damage to 5 for great damage. ^d^Prior stressful life events: scores could range from 0 for no exposure to 5 for high exposure. ^e^Participant’s job status during the pandemic: 0 = job status unchanged (n = 359), 1 = job loss (n = 118). ^f^Spouse’s job status during the pandemic: 0 = job status unchanged (n = 342), 1 = spouse’s job loss (n = 135). ^g^Average family income before the pandemic: scores could range from 1 for very low income to 5 for very high income. ^h^Education: scores could range from 1 for low education to 5 for high education. ^i^Number of children range from 1 to 10.*

## Results

### Bivariate Correlations

#### Stress and Resilience Measures

The results presented in Table 2 reveal positive correlations between the three stress measures (marital, parental, financial). The stress measures were also negatively correlated with the resilience factors.

#### Sociodemographic, Stress, and Resilience Measures

As shown in Table 3, average family income before the pandemic was negatively correlated with each of the three stress measures of stress, and positively correlated with individual resilience. Number of children was positively correlated with parental stress. Education level was negatively correlated with financial stress and family resilience, and positively correlated with individual resilience. Age was positively correlated with individual resilience. Finally, there were no significant correlations of the sociodemographic variables gender, level of religiosity, and marriage duration, with stress levels or resilience factors.

#### Risk, Stress, and Resilience Measures

As shown in Table 3, COVID-19-related financial damage was highly and positively correlated with the three stress measures and negatively correlated with individual resilience. Participant job loss was positively correlated with financial stress. Spouse job loss was positively correlated with marital, parental, and financial stress, and negatively correlated with individual resilience. Stressful life events in the 12 months prior to the pandemic correlated positively with the three stress measures and negatively with individual resilience. Interestingly, exposure to COVID-19 was not correlated with stress levels or with resilience factors.

### Hierarchical Multiple Regression Analyses

Predictors that were significantly correlated with at least one stress variable were included. Table 4 presents the hierarchical regression analysis results for each of the three stress measures. Table 5 presents the dominance analysis results.

#### Marital Stress

The three predictor blocks of predictors explained 26.9% of the variance in marital stress [*F*(9,467) = 19.106, *p* < 0.001]. As hypothesized, the sociodemographic variables explained 3.1% of the variance in marital stress. More specifically, average family income before the pandemic predicted marital stress (β = –0.167, *p* < 0.01). Participants who had a higher average family income before the pandemic reported less marital stress. The number of children variable reached trend level (β = 0.077, *p* = 0.090), while education level (β = 0.028, *p* > 0.10) was not significant.

As predicted, risk factors explained 6.0% of the variance in marital stress, beyond the sociodemographic characteristics [*F change* (4,469) = 7.789, *p* < 0.01], mainly by way of COVID-19-related financial damage (β = 0.249, *p* < 0.0001). Stressful life events within the 12 months prior to COVID-19 reached trend level (β = 0.086, *p* = 0.066).

As predicted, individual and family resilience factors explained 17.7% of the variance in marital stress beyond sociodemographic and risk factor variables [*F change* (2,467) = 56.705, *p* < 0.001]. Taken together, the findings support the hypothesis that resilience factors are better predictors of marital stress than risk factors and sociodemographic characteristics. Moreover, the dominance analysis revealed that while individual resilience explained 6.3% of the variance in marital stress (β = -0.191, *p* < 0.001), family resilience explained 12.5% (β = -0.324, *p* < 0.001). Hence, in reference to the research question, the findings indicate that family resilience better predicted marital stress than did individual resilience.

#### Parental Stress

The three predictor blocks explained 22.2% of the variance in parental stress [*F*(9,467) = 14.83, *p* < 0.001]. As hypothesized, the sociodemographic variables explained 3% of the variance in parental stress. More specifically, a large share of this variance was explained by number of children (β = 0.120, *p* < 0.01) and average family income before the pandemic (β = 0.121, *p* < 0.05).

As predicted, risk factors explained 5.6% of the variance in parental stress, beyond sociodemographic characteristics [*F change* (4,469) = 7.155, *p* < 0.001], mainly by way of COVID-19-related financial damage (β = 0.200, *p* < 0.001) and stressful life events in the 12 months prior to the pandemic (β = 0.113, *p* < 0.05).

As predicted, individual and family resilience explained 13.6% of the variance in parental stress, beyond sociodemographic and risk factors [*F change* (2,467) = 40.919, *p* < 0.001]. Taken together, these findings support the hypothesis that resilience factors are better predictors of parental stress than risk factors and sociodemographic characteristics. Moreover, the dominance analysis indicated that family resilience explained 8.5% of the variance in parental stress (β = -0.257, *p* < 0.001) and individual resilience explained 6.3% (β = -0.200, *p* < 0.001). Hence, in reference to the research question, family resilience better predicted parental stress than did individual resilience.

#### Comparing Marital and Parental Stress

There were many similarities among the predictors that explained variance in marital stress and parental stress. In both cases, resilience factors were the strongest predictors, followed by risk factors (specifically financial damage and stressful events in the 12 months prior to the pandemic), and sociodemographic characteristics (specifically average family income before the pandemic and number of children). Among the resilience factors, family resilience was the better predictor.

#### Financial Stress

The predictor blocks explained 33.7% of the variance in financial stress [*F*(9,467) = 26.408, *p* < 0.001]. As hypothesized, the sociodemographic variables explained 7.5% of the variance in financial stress, mainly by average prior family income (β = –0.266, *p* < 0.001).

As predicted, risk factors explained 21.1% of the variance in financial stress, beyond sociodemographic characteristics [*F change* (4,469) = 34.740, *p* < 0.001], mainly by way of COVID-19-related financial damage (β = 0.490, *p* < 0.001) and stressful life events in the 12 months prior to the pandemic (β = 0.106, *p* < *0.05*).

As predicted, resilience factors explained 5.1% of the variance in financial stress, beyond sociodemographic and risk factors [*F change* (2,467) = 18.000, *p* < 0.001]. Taken together, however, the findings did not support the hypothesis that resilience factors would better predict financial stress compared to others; rather, risk factors were the strongest predictors of financial stress. Moreover, the dominance analysis indicated that individual resilience explained 4.4% of the variance in financial stress (β = –0.164, *p* < 0.0001), while family resilience explained 2.3% (β = –0.115, *p* < 0.05). Hence, in reference to the research question, individual resilience better predicted financial stress than did family resilience.

#### Comparing Marital, Parental, and Financial Stress

The predictors of variance in marital and parental stress differed considerably from the predictors of financial stress. For both marital and parental stress, resilience factors (mainly family resilience) were the strongest predictors, followed by risk factors and sociodemographic characteristics, respectively. For financial stress, risk factors (mainly COVID-19-related financial damage and prior stressful life events) were the strongest predictors, followed by sociodemographic characteristics and resilience factors (mainly individual resilience).

## Discussion

The COVID-19 pandemic is an unprecedented event that has threatened the physical and mental health of many individuals, as well as the stability and functioning of families worldwide. The current study aimed to determine how marital, parental, and financial stress during the pandemic are related to various sociodemographic characteristics, risk factors, and resilience factors among married Palestinian individuals in Israel. The contrasting roles of the three groups of predictors were examined, to test the hypothesis that resilience factors would better predict stress than risk factors and sociodemographic characteristics. In addition, given the collectivistic nature of Palestinian society in Israel, in which the influence of family resources is highly significant, it was interesting to explore whether family resilience would better predict stress than did individual resilience, particularly when the outcome variables involved family functioning.

The results confirmed the hypotheses regarding the contributions of the three groups of predictors (sociodemographic characteristics, risk factors, and resilience factors) to the three stress measures. The fourth hypothesis was confirmed with respect to both marital and parental stress, but not financial stress. That is, resilience factors were the strongest predictors of marital and parental stress, followed by risk factors and then sociodemographic characteristics. Meanwhile, risk factors, and more specifically COVID-19-related financial damage and stressful events in the 12 months prior to the pandemic, were the strongest predictors of financial stress, followed by sociodemographic characteristics and then resilience factors. The study findings are discussed in detail below.

### Sociodemographic Predictors of Stress

As predicted, several socioeconomic characteristics predicted the three stress measures. Average family income before to the pandemic negatively predicted all three measures, such that higher income was associated with lower stress. Greater numbers of children predicted higher marital and parental stress levels, and education level was negatively correlated with financial stress. These findings corroborate previous work indicating that sociodemographic characteristics, including socioeconomic status and education level are associated with various outcome variables in the context of disasters and crises in general (Palmieri et al., 2008) and the COVID-19 pandemic specifically (Kimhi et al., 2020b,c). The current findings are supported by Hobfoll’s conservation of resources theory (1989, 1998), which suggests that sociodemographic variables are predictors of stress, and specifically that those with fewer social or financial resources are more negatively affected by stressors.

Stress levels in the current study were not correlated with age, gender, level of religiosity, or marriage duration. These findings are in line with some of the work already done during the current pandemic (Kharshiing et al., 2021), though some studies indicate that female gender and younger age are associated with higher anxiety (Barzilay et al., 2020) and with sense of danger and distress (Kimhi et al., 2020c).

### Risk Factors as Predictors of Stress

Also in accordance with the hypotheses, risk factors predicted the three stress measures, beyond the contribution of sociodemographic characteristics. Financial damage due to the pandemic, and stressful life events in the 12 months prior to its start were the key predictors. These findings are consistent with previous research indicating that financial difficulties related to COVID-19 predict generalized anxiety and depression (Barzilay et al., 2020), greater sense of danger and symptoms of distress (Kimhi et al., 2020b,c; Shanahan et al., 2020), and emotional distress (Shanahan et al., 2020). Pre-pandemic stressful life events were also associated with emotional distress during the pandemic (Shanahan et al., 2020). These results indicate that individual characteristics and environmental stressors before or after an event can impact potential trauma more than the objective nature of the event itself (Galatzer-Levy et al., 2018).

Exposure to COVID-19 was not significantly related to any of the stress measures, in accordance with a previous study indicating that health risks to self and others during the pandemic were only weakly associated with stress (Shanahan et al., 2020). Shanahan and colleagues explained this result based on the fact that only a small percentage of participants had been exposed to the most traumatic aspects of the pandemic (e.g., death of a loved one or hospitalization due to COVID-19). Similarly, in the current study, only a small percentage of participants, or their family members, friends, and acquaintances, had been infected with the coronavirus, quarantined, or known someone who died as a result of infection.

Participant job loss was positively correlated only with financial stress, supporting other studies (Shanahan et al., 2020). Meanwhile, spouse job loss was positively correlated with all three stress measures. It appears that when participants lost their own jobs, they were able to function sufficiently at home and maintain acceptable levels of marital and parental stress. When their spouses lost jobs, however, these types of stress rose alongside financial stress, presumably due to the resultant lifestyle changes. It is also noteworthy in this context that 70% of the study sample identified as female. Among Palestinian households in Israel, women generally earn less than men and wives are still much less likely to be the primary wage earners (Arar and Mustafa, 2009). This could mean that the financial damage (and the related stress) incurred if they lost their jobs was less substantial than when their husbands lost their jobs.

Still, when participant job loss and spouse job loss were added to the regression analysis, together with risk and resilience factors, they contributed little to the percentage of explained variance in the stress measures. COVID-19-related financial damage explained a large part of the variance in stress, masking other significant correlations that were found. Once again, the findings are consistent with Hobfoll’s conservation of resources theory (1989, 1998), which suggests not only that the existence of personal, social, and material resources is important but also that the ability to maintain these resources following stressful events will be a major predictor of lower stress.

### Resilience Factors as Predictors of Stress

The two types of resilience, as hypothesized, negatively predicted marital, parental, and financial stress, beyond the effects of sociodemographic characteristics and risk factors. Family resilience has previously been shown to improve coping with various crises, including a major epidemic (Chang et al., 2015; Neo et al., 2016) and political violence (Khamis, 1998; Sousa et al., 2013), as well as to decrease the risk of poor prenatal and postnatal mental health (Qutteina et al., 2018) and dwelling on the terrible nature of events (Don and Mickelson, 2012).

The finding that individual resilience had a negative relationship with stress is consistent with research indicating a role for resilience factors in coping with crises (Suedfeld, 2015; Galatzer-Levy et al., 2018; Eshel et al., 2019), including the COVID-19 pandemic (Kimhi et al., 2020b,c; Nair et al., 2020; Prime et al., 2020). In these previous studies, high levels of resilience were associated with fewer worries and less generalized anxiety and depression.

### Comparing the Roles of Sociodemographic, Risk, and Resilience Variables

As hypothesized, the strongest predictors of both marital and parental stress were resilience factors, predominantly family resilience, followed by risk factors and then sociodemographic characteristics. In contrast, risk factors, particularly pandemic-related financial damage and prior stressful life events, were the strongest predictors of financial stress. They were followed by sociodemographic characteristics and then resilience factors, particularly individual resilience.

The precedence of resilience factors in predicting marital and parental stress lend empirical support to previous assumptions that the extent to which COVID-19 would adversely impact individuals and families would depend on other related factors in their lives, and particularly on family resilience prior to the pandemic (Chen and Bonanno, 2020; Prime et al., 2020). Family and individual resilience are protective factors that accentuate the ability to function under difficult conditions (Chen and Bonanno, 2020; Pietromonaco and Overall, 2021). In the current study, this “buffering effect” was evident in the measures of both family and individual resilience. Extending previous studies showing that resilience predicted individual distress levels during the COVID-19 pandemic, the current findings show a similar pattern in the context of family functioning.

Resilience factors did not show priority in predicting financial stress. Rather, risk factors, and particularly pandemic-related financial damage and prior stressful life events, were better predictors. Previous work has indeed suggested that when financial conditions are disrupted, resilience is not sufficient to counteract financial stress. These differences in the pattern of predictors can also be considered in the context of family systems theory. Namely, while marital and parental stress directly involve difficulties in family relationships and subsystems, financial stress is generally an external effect on the family system as a whole. As such, it is likely that resilient relationships helped weather unfavorable circumstances within the family system or subsystems as a consequence of COVID-19 (Prime et al., 2020), but had less of an impact under financial stress. This highlights the importance of governmental financial support during unexpected humanitarian crises (Kimhi et al., 2020c; Prime et al., 2020).

Regarding the comparison between individual and family resilience, the latter appeared to be a better predictor of marital and parental stress. These findings likely reflect the coping patterns of a collectivistic society. In the collectivistic cultural context examined here, it appears that the traditional value of external family support encompassed the nuclear family, highlighting the importance of nuclear family resilience during a crisis that limited extended family support. It is also possible that COVID-19 is perceived as a “shared challenge” (Simon et al., 2005) because it threatens the entire family. Under such conditions, individuals might be more likely to derive combined strength from solidarity with family members (Neo et al., 2016), especially with respect to marital and parental functioning. This explanation needs further empirical support.

### Limitations

The findings and conclusions of this study should be considered in the context of several limitations. First, certain inherent biases were introduced due to the sampling method employed, as all participants had access to and were users of social media, and chose to complete an online survey, possible resulting in a selection bias. In particular, it is possible that the sampling method contributed to the bias toward participants who identified as women, who made up about 70% of the final sample. This might have affected the results and could limit their generalizability of the results, although a large sample of participants with a broad sociodemographic range was included. Second, the study relied on self-report questionnaires, which are subjective by definition and affected by various biases.

Third, the study was cross-sectional, making it impossible to determine causative relationships between the variables. In addition, the findings are specific to the time frame beginning with the outbreak of the pandemic and ending around end of the first lockdown. Future studies are needed to evaluate whether the reported associations are maintained longitudinally, beyond the acute stage of the outbreak, given that stress is likely to increase as mortality rates rise and economic burdens deepen. Longitudinal studies could reveal exacerbation of negative consequences, as well as recovery and even post-traumatic growth.

Finally, the study focused on married individuals in Israel’s Palestinian population, a collectivistic minority society in which families function as teams during the stress-and-coping process. Particularly with respect to the comparative roles of individual versus family resilience factors, the current findings might not be generalizable to more individualistic cultures and non-minority cultures. Furthermore, the Palestinian study population was not compared to the Jewish majority, which precludes the ability to attribute the study findings specifically to minority status and sociopolitical characteristics. Future studies should address these concerns.

### Conclusion and Implications

This study increases our understanding of variables related to marital and parental stress during the COVID-19 pandemic. The findings can be applied in designing interventions for couples and families, focused on developing and maintaining resilience factors to counteract crisis-related negative effects and risk factors, and enable healthy marital and parental functioning.

The Palestinian society in Israel has rarely been studied in the context of family functioning. Reliance on family resilience is known to vary as a function of cultural values and beliefs (Walsh, 1998; Patterson, 2002). In the collectivistic cultural context examined here, it appears that addressing family resilience in therapeutic interventions among couples would be beneficial. Namely, couples should be made aware of the benefits of family resilience for all family members, including young children and adolescents, and of the potentially negative consequences of its absence.

Previous studies have addressed methods for increasing resilience in the process of couples and family therapy by raising awareness of its importance and setting related goals. Chang et al. (2015), for example, identified specific components of resilience to work toward, such as effective communication, control of negative feelings, and shared belief systems. A specific therapeutic method that can be used is this context is Greenberg and Goldman’s (2008) Emotion-Focused Therapy for Couples (CFT-C), which sets mutual affect regulation as its goal. Based on systematic and experiential-humanistic approaches, this method is a basis for acquiring additional skills that promote relationship functioning.

We should also work to promote individual resilience, through personal development and independence, coping skills, and improved emotional and behavioral regulation. Future work in other cultural contexts might shed light on the relative importance of addressing family and individual resilience in different groups.

Finally, the current study extends research demonstrating variability in responses to traumatic events at both the individual (Tedeschi and Calhoun, 1996; Bonanno, 2005), and family (Li et al., 2018) levels. Namely, the findings shed light on the interplay between sociodemographic characteristics, risk factors, and individual and familial resilience factors, which come together to explain why some individuals and couples exhibit positive coping, recovery, and decreased burnout, while others experience the opposite.

## Data Availability Statement

The raw data supporting the conclusions of this article will be made available by the author, without undue reservation.

## Ethics Statement

The study was reviewed and approved by the Ethics Committee of Zefat Academic College. The participants provided their written informed consent to participate in this study.

## Author Contributions

The author confirms being the sole contributor of this study and has approved it for publication.

## Conflict of Interest

The author declares that the research was conducted in the absence of any commercial or financial relationships that could be construed as a potential conflict of interest.

## Publisher’s Note

All claims expressed in this article are solely those of the authors and do not necessarily represent those of their affiliated organizations, or those of the publisher, the editors and the reviewers. Any product that may be evaluated in this article, or claim that may be made by its manufacturer, is not guaranteed or endorsed by the publisher.

## Footnotes

1
https://surveys.cbs.gov.il/Survey/surveyE.htm

2
https://www.R-project.org/
